# Perceived social support, perceived stress, and athlete burnout among competitive athletes: a mediation model

**DOI:** 10.3389/fpsyg.2026.1929837

**Published:** 2026-10-29

**Authors:** Weihan Yuan, Guangshu Wu, Guihong Wang

**Affiliations:** 1 Shenyang Sport University, Shenyang, Liaoning, China; 2 Tomsk State University, Tomsk, Russia

**Keywords:** athlete burnout, competitive athletes, cross-sectional study, general perceived stress, perceived social support

## Abstract

**Background:**

Perceived social support is an important psychosocial resource for competitive athletes, while perceived stress is closely associated with athlete burnout. Drawing on conservation of resources theory and the cognitive-transactional model of stress, this study examines the relationships among perceived social support, perceived stress, and athlete burnout.

**Objectives:**

This study aims to investigate the associations among perceived social support, perceived stress, and athlete burnout in competitive athletes, with particular attention to the statistical mediating role of perceived stress.

**Results:**

Using cluster sampling, questionnaire data were collected from 252 competitive athletes across various sports, including track and field, swimming, and football. The results showed that perceived social support was negatively correlated with perceived stress (*r* = -0.478, *p* < 0.01) and athlete burnout (*r* = -0.282, *p* < 0.01), whereas perceived stress was positively correlated with athlete burnout (*r* = 0.421, *p* < 0.01). Mediation analysis revealed a significant indirect association between perceived social support and athlete burnout through perceived stress (*B* = -0.161, 95% CI [-0.240, -0.093]). The direct association was not statistically significant after accounting for perceived stress (*B* = -0.095, *p* = 0.109).

**Conclusion:**

Perceived social support is associated with lower levels of athlete burnout, both through its overall negative association with burnout and through a significant statistical indirect pathway involving perceived stress. These findings highlight the importance of strengthening athletes’ social support systems and addressing perceived stress in competitive sports settings. Given the cross-sectional design, the findings should be interpreted as associations rather than evidence of causal mediation.

## 1 Introduction

Against the backdrop of the “Healthy China” and “Sports Powerhouse” strategies, the balanced physical and mental development of athletes has become a key component of competitive athlete training. The “Healthy China 2030” Outline calls for strengthening athletes’ cultural education and psychological counseling to promote the coordinated development of competitive performance and mental health (Central Committee of the Communist Party of China, State Council of the People’s Republic of China, 2016). The General Administration of Sport of China further emphasized mental health services and routine psychological support for athletes in the “14th Five-Year Plan” for Competitive Sports (General Administration of Sport of China, 2021). In recent years, domestic research has begun to explore the athlete mental health support system from perspectives such as service philosophy, organizational models, and professional collaboration (Shi and Wang, 2024; Wang and Cao, 2022), while international position statements and consensus documents have also emphasized prevention, referral, multidisciplinary collaboration, and the athlete’s full-cycle development (Moesch et al., 2018; Reardon et al., 2019). Consequently, psychological support for sports teams is no longer limited to pre-competition emergency response or problem resolution but has gradually become an ongoing component of training and competition management. Identifying measurable psychosocial factors associated with athlete burnout helps translate macro-level mental health goals into testable issues at the individual level.

Competitive sports are characterized by high training loads, dense competition schedules, clear evaluation criteria, and significant career uncertainty. Athletes are constantly immersed in an environment where performance goals, training demands, and lifestyle management are intertwined; consequently, their psychological state is not limited to short-term emotional fluctuations. Athlete burnout is a relatively typical chronic psychological adaptation issue in competitive sports, primarily comprising three dimensions: emotional and physical exhaustion, reduced sense of accomplishment, and devaluation of sport (Raedeke and Smith, 2001; Chen and Zhou, 2007). It is not the same construct as short-term mental fatigue that occurs after sustained cognitive tasks; the latter emphasizes temporary cognitive exhaustion, whereas athlete burnout involves changes in the meaning of sport, the experience of achievement, and the evaluation of long-term commitment. Relevant reviews suggest that burnout is the result of the combined effects of factors such as stress, motivation, relational environments, and insufficient recovery (Gustafsson et al., 2017); meta-analyses across time indicate that some burnout symptoms are on the rise (Madigan et al., 2022), while systematic reviews show that athlete burnout is associated with poorer mental health and well-being (Glandorf et al., 2025). Existing longitudinal evidence remains limited, and the directionality and interactions among variables require further testing through repeated-measure studies (Dišlere et al., 2025).

In the practice of psychological support for sports teams, social support has consistently been regarded as a crucial external resource. The support received by elite athletes may come from family, friends, and significant others, as well as from coaches and teammates within the sports context (Rees and Hardy, 2000; Zimet et al., 1988). What can truly be directly observed in research is often athletes’ subjective perceptions of this support. Existing meta-analyses show that social support and the fulfillment of relational needs are generally negatively associated with athlete burnout, while negative social interactions are associated with higher levels of burnout (Pacewicz et al., 2019). Studies involving weightlifters, college athletes, and multi-sport athletes have also found that higher perceived support is typically accompanied by lower burnout or better mental health (Shang and Yang, 2021; Gray et al., 2024; Murray et al., 2023; Zhao et al., 2022); Studies of Chinese athletes further suggest that the relationship between organizational stressors and burnout may be influenced by differences in social support (Wu et al., 2016). This evidence supports viewing perceived social support as an important factor related to athlete burnout, but it does not indicate that every source of support plays the same role. The scale used in this study does not include separate subscales for support from coaches, teammates, and the organization; therefore, the analysis focuses on overall perceived social support.

Perceived stress reflects an individual’s overall assessment of the unpredictability, uncontrollability, and overwhelming nature of recent life events (Cohen et al., 1983; Yang and Huang, 2003). It emphasizes how individuals interpret environmental demands rather than the sheer number of objective events. The PSS-14 measures general stress experiences over the past month, which may be influenced by training and competition but may also include stressors such as academics, interpersonal relationships, family, and life management; therefore, it cannot be equated with sport-specific stress. Existing systematic reviews and meta-analyses have shown a consistent positive relationship between athlete stress and burnout (Lin et al., 2022); Studies employing stress perception measures have also found that higher levels of perceived stress are associated with greater burnout (Gustafsson and Skoog, 2012; Olsson et al., 2022; Chen et al., 2011). Based on Resource Conservation Theory and the Cognitive Transactional Stress Model, this study examines the relationship between perceived social support and athlete burnout while further investigating the statistical indirect effect mediated by general stress perception. Due to the lack of time-series data, this analysis is used to present the statistical structure of the three variables at a single point in time and does not serve to establish causal sequence.

## 2 Theoretical framework and research hypotheses

### 2.1 Theoretical foundation

#### 2.1.1 Research variables

This study involves three interrelated but distinct constructs. Perceived social support refers to athletes’ subjective evaluation of the support provided by family, friends, and significant others; its core lies not in the objective frequency of supportive behaviors, but in whether individuals feel they can receive understanding, companionship, and assistance (Rees and Hardy, 2000; Zimet et al., 1988). In the actual questionnaire, some “significant others” items cited coaches, relatives, and teammates as examples; however, since the scale does not include source-specific subscales, this study uses only the overall perceived social support score. General perceived stress refers to an individual’s overall experience of life events over the past month that are unpredictable, difficult to control, and exceed their coping capacity (Cohen et al., 1983; Yang and Huang, 2003); it does not refer specifically to training load or competition results. Athlete burnout was measured using the Athlete Burnout Questionnaire, which includes three dimensions: emotional and physical exhaustion, reduced sense of accomplishment, and devaluation of sport (Raedeke and Smith, 2001; Chen and Zhou, 2007). These three dimensions correspond, respectively, to the subjective perception of external psychosocial resources, recent stress experiences, and the chronic adaptation outcomes in a sports context, and are clearly distinguished theoretically.

#### 2.1.2 Theoretical basis

Resource Conservation Theory posits that individuals have a fundamental tendency to acquire, maintain, and protect important resources; when resources are continuously threatened, depleted, or expended without being adequately replenished, individuals are more likely to experience stress and exhaustion (Hobfoll, 1989; Hobfoll et al., 2018). For competitive athletes, time, physical fitness, a sense of control, positive relationships, and available support can all serve as important resources. Training and competition themselves do not necessarily lead to burnout; the key lies in whether athletes are chronically in a state of high investment, low recovery, or insufficient resource replenishment. Perceived social support reflects athletes’ assessment of the availability of interpersonal resources and is therefore theoretically associated with lower emotional exhaustion, higher feelings of accomplishment, and less devaluation of sport. Research on social relationships and athlete burnout generally supports this negative association (Pacewicz et al., 2019; Shang and Yang, 2021; Gray et al., 2024; Murray et al., 2023; Zhao et al., 2022; Wu et al., 2016); however, cross-sectional correlations can only demonstrate the co-variation between perceived support and burnout at a single point in time.

The cognitive-transactional stress model emphasizes that stress is not a direct replica of environmental stimuli, but rather a subjective experience resulting from an individual’s evaluation of situational demands and their own coping resources (Lazarus and Folkman, 1984). When individuals perceive demands to exceed available resources, they are more likely to experience feelings of uncontrollability and overload. The stress buffering model further proposes that social support may be associated with lower stress responses by improving resource evaluations, providing problem-solving cues, or alleviating feelings of isolation (Cohen and Wills, 1985). In research on athlete burnout, coping resources and stress processes are also frequently placed within the same analytical framework (Raedeke and Smith, 2004). Based on this, the present study examines the relationships between perceived social support and burnout, general stress perception and burnout, and social support and stress perception, respectively. These three pathways constitute a statistical mediation model but are not presupposed as established causal chains.

### 2.2 Research hypotheses

#### 2.2.1 Understanding social support and athlete burnout

Social support provides athletes with emotional care, information exchange, and a sense of belonging, serving as a relatively stable psychosocial resource in competitive settings. Both meta-analyses and studies across various sports have shown that social support is generally negatively associated with athlete burnout (Pacewicz et al., 2019; Shang and Yang, 2021; Gray et al., 2024; Murray et al., 2023; Zhao et al., 2022; Wu et al., 2016). This association may manifest in the fact that athletes who feel supported are less likely to experience feelings of isolation and resource depletion when faced with sustained commitment and performance evaluation. It should be noted that this study measures perceived social support as a whole, rather than the independent effects of support from coaches, teammates, or family; therefore, the hypothesis applies only to the overall score. Based on this, we propose:

*H1*: Perceived social support is significantly negatively correlated with athlete burnout.

#### 2.2.2 Understanding social support and general perceived stress

General perceived stress reflects an individual’s overall assessment of the unpredictability, uncontrollability, and overwhelming nature of life events over the past month (Cohen et al., 1983; Yang and Huang, 2003). As a perceived interpersonal resource, social support may enhance an individual’s sense of control over difficult situations and may also provide external conditions for problem-solving and emotional regulation. Research on social support among elite athletes and stress buffering theory both suggest that greater support is often associated with lower levels of perceived stress (Rees and Hardy, 2000; Wu et al., 2016; Cohen and Wills, 1985). However, since the PSS-14 does not distinguish between sources of stress, this study can only examine the relationship between perceived social support and general stress perception overall; the results cannot be limited to training or competition-related stress. Based on this, Hypothesis is proposed:

*H2*: Perceived social support is significantly negatively correlated with general stress perception.

#### 2.2.3 General perceived stress and athlete burnout

A persistently high level of perceived stress means that individuals more frequently perceive life events as unpredictable, uncontrollable, or beyond their capacity to handle. When this experience persists over the long term, it may be accompanied by emotional and physical exhaustion, reduced sense of accomplishment, and a negative evaluation of the value of their commitment to sports. International reviews, meta-analyses, and studies of athlete samples all support a positive relationship between perceived stress and athlete burnout (Lin et al., 2022; Gustafsson and Skoog, 2012; Olsson et al., 2022; Chen et al., 2011); Studies of Chinese athletes have also examined the relationship between organizational stressors and athlete burnout, including the potential moderating roles of social support and mental toughness (Wu et al., 2016; Wu, 2022). Since this study employed the General Perceived Stress Scale, the results reflect the correlation between overall stress experience and athlete burnout, rather than the effect of stress specific to a particular sport. Based on this, we propose:

*H3*: General perceived stress is significantly positively correlated with athlete burnout.

#### 2.2.4 Statistical indirect effects of general perceived stress

Building on the three relationships described above, this study further examines whether there is a statistical indirect effect between perceived social support and athlete burnout mediated by general stress perception. Theoretically, higher perceived social support may co-occur with lower perceived stress, and lower perceived stress may be associated with lower athlete burnout, thereby forming a statistical indirect path. Previous studies on athletes have observed similar relationships among stress resources, general perceived stress, and burnout (Gustafsson and Skoog, 2012; Olsson et al., 2022; Chen et al., 2011; Raedeke and Smith, 2004). However, cross-sectional mediation analyses cannot confirm the sequence of variables nor rule out the possibility that athlete burnout may, in turn, influence their perceived support and stress evaluations. Therefore, H4 tests only the statistical indirect effect. Based on this, H4 is proposed:

*H4*: There is a significant statistical indirect effect between perceived social support and athlete burnout mediated by general stress perception.

## 3 Subjects and methods

### 3.1 Subjects

The competitive athletes included in this study comprised athletes currently undergoing systematic, cyclical training with the goal of participating in official competitions at the provincial level or higher. The study population included both athletes holding a “Technical Grade Certificate for Athletes” of Level 2 or higher and registered athletes affiliated with qualified training organizations, such as sports teams directly under provincial (municipal) sports bureaus, university sports teams, and youth sports clubs. Therefore, the “Other” sports grade category listed in Table 1 does not necessarily imply that an athlete did not meet the inclusion criteria. A multistage cluster sampling method was employed to recruit athletes from 32 training teams across 25 provincial-level administrative regions, yielding a total of 268 raw records. Data verification revealed that 16 of these records contained all “1”s across 48 variables, with no response variation; these were therefore excluded as systematically missing records. Ultimately, 252 records were included, resulting in a response rate of 94.03%. The sample covered different genders, athletic levels, team tiers, and sports; however, no inferences regarding national representativeness are drawn based on this. The age options “20–23 years” and “23–28 years” in the original questionnaire overlapped at age 23; since exact ages were not recorded, the original classifications were retained in the analysis.

**Table 1:** Descriptive analysis of basic information of athletes.

Variable	Category	Frequency	Percentage*/%*
Gender	Male	140	55.6
	Female	112	44.4
Team level	National-level Youth Sports Club	124	49.2
	College Sports Teams	79	31.3
	Provincial Team	46	18.3
	National Team	3	1.2
Competitive level	Level 2 Athlete	77	30.6
	Level 1 Athlete	86	34.1
	Master of Sports	9	3.6
	Other	80	31.7
Age	15 and under	120	47.6
	16 to 19	43	17.1
	20 to 23	81	32.1
	23 to 28	7	2.8
	29 and over	1	0.4
Years of experience	Less than 3 years	69	27.4
	4–7 years	79	31.3
	8–10 years	34	13.5
	10 years or more	70	27.8
Number of training sessions per week	Twice a week or less	12	4.8
	Three times a week	35	13.9
	Four times a week	34	13.5
	Five times a week	42	16.7
	Six times a week or more	129	51.2
Duration of each training session	Less than 2 h	119	47.2
	2–3 h	108	42.9
	3–4 h	17	6.7
	4–5 h	3	1.2
	5 h or more	5	2
Sports	Swimming	32	12.7
	Track and Field	27	10.7
	Soccer	24	9.5
	Basketball	23	9.1
	Badminton	16	6.3
	Volleyball	16	6.3
	Judo	10	4.0
	Fencing	9	3.6
	Canoeing (Single)	9	3.6
	Shooting	9	3.6
	Short Track Speed Skating	8	3.2
	Speed Skating	8	3.2
	Weightlifting	8	3.2
	Table Tennis	8	3.2
	Beach Volleyball	8	3.2
	Taekwondo	8	3.2
	Boxing	8	3.2
	Wrestling	7	2.8
	Artistic Gymnastics	7	2.8
	Rowing (Four-Man)	7	2.8

### 3.2 Measuring tools

This study used a questionnaire survey to collect data; the questionnaire included basic personal information and three psychological assessment tools.

The Perceived Social Support Scale is based on the Multidimensional Scale of Perceived Social Support developed by Zimet et al. (1988) and primarily covers support from family, friends, and significant others. The actual questionnaire uses a 5-point rating scale, with some items regarding significant others providing examples such as coaches, relatives, and teammates. A higher scale score indicates a higher overall level of perceived social support; Cronbach’s α is 0.947.

The “Athlete Burnout Questionnaire” is based on the Athlete Burnout Questionnaire developed by Raedeke and Smith and its Chinese-language adaptation (Raedeke and Smith, 2001; Chen and Zhou, 2007). The scale comprises three dimensions—emotional/physical exhaustion, reduced sense of accomplishment, and devaluation of sport—with a total of 15 items. Items 1 and 14 are reverse-scored and use a 5-point frequency rating scale. Higher scores indicate higher levels of athlete burnout; Cronbach’s α was 0.913.

The Perceived Stress Scale (PSS) utilized the PSS-14 developed by Cohen et al. and its Chinese version (Cohen et al., 1983; Yang and Huang, 2003), which is used to assess an individual’s general perceived stress over the past month. The scale consists of 14 items, of which items 4, 5, 6, 7, 9, 10, and 13 are reverse-scored items, using a 5-point frequency rating scale. A higher score indicates a higher level of general perceived stress; Cronbach’s α was 0.822.

### 3.3 Data processing and statistical analysis

Statistical analysis was performed using SPSS and PROCESS Model 4 (Hayes, 2018). First, demographic characteristics, means, standard deviations, skewness, kurtosis, and Pearson correlation coefficients were calculated, and a diagnosis of multicollinearity was conducted. Subsequently, the statistical indirect effect of general perceived stress on the relationship between perceived social support and athlete burnout was tested, using 5,000 Bootstrap repetitions to calculate the 95% confidence interval. All regression coefficients are reported as unstandardized coefficients B. Given differences in the sample regarding gender, team level, sport level, age, and training characteristics, these variables were further included in a sensitivity analysis of covariates, and HC3 heteroscedasticity-robust standard errors were used.

Participants were recruited from 32 training teams; athletes within the same team may have had intra-group correlation. The final analysis file did not retain individual team identifiers; therefore, it was not possible to reliably calculate the ICC or use team-cluster-robust standard errors. This data structure may affect the precision of standard errors and confidence intervals; these limitations are taken into account in the interpretation of the results.

## 4 Results and analysis

### 4.1 Test for deviation from the common method

This study employed Harman’s one-factor test to assess common-method bias (Harman, 1976). All item items were included in an unrotated principal component analysis, which identified three factors with eigenvalues greater than 1. The first factor explained 21.865% of the variance, falling short of the empirical threshold of 40%. While this result does not indicate that a single factor is dominant, it does not fully rule out common-method bias.

### 4.2 Descriptive statistics and distribution characteristics of each variable

Descriptive statistics were calculated for the scores of 252 participants on each study variable. The mean score for general perceived stress was 2.593, with a standard deviation of 0.607, a skewness of −0.092, and a kurtosis of 0.013; the mean score for perceived social support was 3.900, with a standard deviation of 0.751, a skewness of −0.303, and a kurtosis of 0.052; the mean score for athlete burnout was 2.539, with a standard deviation of 0.683, a skewness of 0.554, and a kurtosis of 1.017. Both the Kolmogorov–Smirnov and Shapiro–Wilk tests yielded *p*-values < 0.001, indicating that the data deviated from a strict normal distribution; therefore, normality could not be assumed. Given that the skewness and kurtosis did not show extreme deviations, and that the indirect effects were tested using the Bootstrap method, the subsequent analysis proceeded as planned.

### 4.3 Understanding the correlation matrix and diagnosis of multicollinearity among social support, perceived general stress, and athlete burnout

Perceived social support was significantly negatively correlated with general perceived stress (*r* = −0.478, *p* < 0.01); general perceived stress was significantly positively correlated with athlete burnout (*r* = 0.421, *p* < 0.01); and perceived social support was significantly negatively correlated with athlete burnout (*r* = −0.282, *p* < 0.01). The tolerance values for both predictor variables were 0.772, and the VIF values were both 1.296, indicating no severe multicollinearity. Correlation analysis reflects linear relationships among variables and is not used to determine the direction of causality (see Table 2).

**Table 2:** Correlation matrix of perceived social support, general perceived stress, and athlete burnout (*N* = 252).

Variables	Average	Standard deviation	General perception of stress	Understanding social support	Athlete burnout
General perceived stress	2.593	0.607	1		
Perceived social support	3.900	0.751	−0.478^**^	1	
Athlete burnout	2.539	0.683	0.421^**^	−0.282^**^	1

**p < 0.01.

### 4.4 Cross-sectional statistical mediation analysis

The PROCESS Model 4 was used to test the statistical indirect effects of general pressure perception; 5,000 bootstrap repetitions were conducted, and 95% confidence intervals were calculated. Since the three variables were measured at the same point in time, this analysis describes a cross-sectional statistical relationship and is not intended to establish temporal sequence or causal mediation.

Tables 3, 4 present the results of the mediation analysis. The overall association between perceived social support and athlete burnout was significant (B = −0.256, SE = 0.055, t = −4.643, *p* < 0.001), supporting H1; perceived social support was significantly negatively associated with general perceived stress (B = −0.386, SE = 0.045, t = −8.598, *p* < 0.001), supporting H2; there was a significant positive association between perceived general stress and athlete burnout, with (B = 0.417, SE = 0.073, t = 5.690, *p* < 0.001), supporting H3. After controlling for general stress perception, the direct association between perceived social support and athlete burnout was B = −0.095, SE = 0.059, t = −1.609, *p* = 0.109, which did not reach statistical significance. The estimated indirect effect was −0.161, BootSE = 0.038, 95% CI [−0.240, −0.093]; the confidence interval did not include 0, supporting H4. This result reflects an “indirect-only” pattern, which under traditional classification could be termed a complete mediation; however, the cross-sectional design does not support a causal mediation explanation (see Figure 1).

**Table 3:** Cross-sectional mediation regression results (*N* = 252).

Variables	Athlete burnout (after accounting for general perceived stress)			A general model of athlete burnout			General model of stress perception		
	B	Standard error	t	B	Standard error	t	B	Standard error	t
Perception of social support	−0.095	0.059	−1.609	−0.256	0.055	−4.643^***^	−0.386	0.045	−8.598^***^
General perceived stress	0.417	0.073	5.690^***^						
*R^2^*	0.185			0.079			0.228		
*F*	28.320^***^			21.558^***^			73.926^***^		

B is the unstandardized coefficient; ***p < 0.001. For the direct association, B = −0.095, t = −1.609, *p* = 0.109; the result is not significant.

**Table 4:** Total, direct, and statistical indirect effects.

Effects	Effect size	SE/BootSE	Lower limit of the 95% CI	Upper limit of the 95% CI	Statistical conclusions
Statistical indirect effects	−0.161	0.038	−0.240	−0.093	Significant
Direct association	−0.095	0.059	−0.212	0.021	Not Significant
Total association	−0.256	0.055	−0.365	−0.148	Significant

Bootstrap sampling was performed 5,000 times.

**Figure 1:**
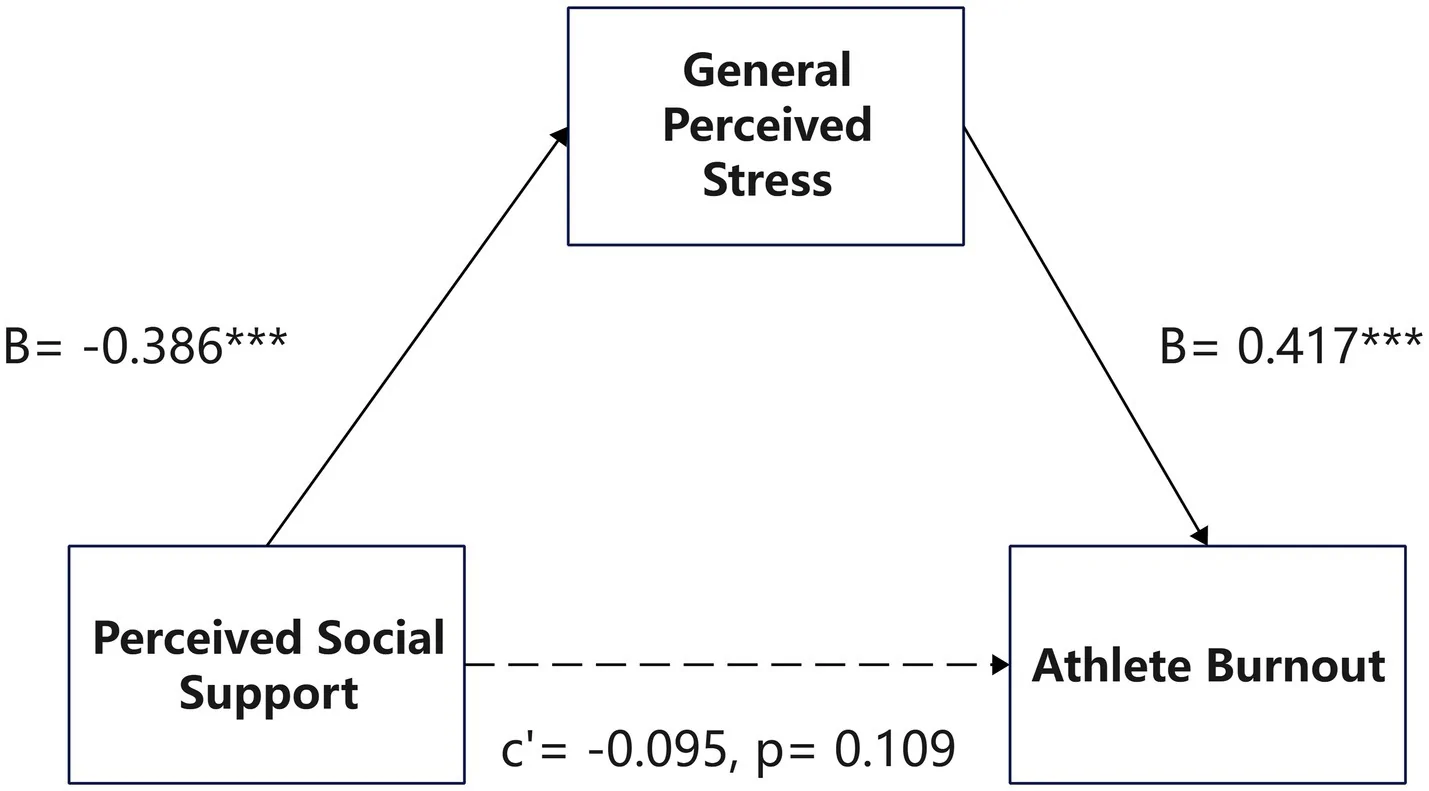
Cross-sectional mediation model of perceived social support, general perceived stress, and athlete burnout. The path coefficients in the figure are unstandardized coefficients B; dashed lines indicate that the direct association is not significant; the total association is c = −0.256, *p* < 0.001.

The three R^2^ values correspond to different regression equations. The model in which perceived social support predicts general stress perception has R^2^ = 0.228, *F* = 73.926, *p* < 0.001; the model in which perceived social support predicts athlete burnout has R^2^ = 0.079, *F* = 21.558, *p* < 0.001; the outcome model in which perceived social support and general stress perception jointly predict athlete burnout had R^2^ = 0.185, *F* = 28.320, *p* < 0.001. Given that the direct associations were not statistically significant, this study does not further report the proportions of the total effect attributable to direct and indirect effects.

### 4.5 Sensitivity analysis adjusted for covariates

After further controlling for gender, team level, athletic level, age group, years of training, number of weekly training sessions, and duration of a single training session, social support was still significantly negatively associated with general perceived stress, with B = −0.362, HC3 SE = 0.047, *p* < 0.001; General perceived stress remained significantly positively associated with athlete burnout (B = 0.365, HC3 SE = 0.090, *p* < 0.001). The adjusted total association between perceived social support and athlete burnout was B = −0.222, HC3 SE = 0.077, *p* = 0.004; the direct association after including perceived general stress was B = −0.090, HC3 SE = 0.087, *p* = 0.299. After 5,000 bootstrap repetitions, the adjusted statistical indirect effect was −0.132, 95% CI [−0.206, −0.066]. After adjusting for covariates, the statistical indirect effect remained significant while the direct association was not significant; the pattern of findings was consistent with that of the unadjusted analysis.

## 5 Discussion

### 5.1 The relationship between athlete burnout and perceived social support and general perceived stress

The results show that perceived social support is significantly and negatively associated with general stress perception. This finding is consistent with the stress buffering perspective and existing research on social support among athletes (Rees and Hardy, 2000; Gray et al., 2024; Wu et al., 2016; Cohen and Wills, 1985). Interpersonal support may coexist with a greater perceived availability of coping resources and a lower sense of helplessness; however, training and competition do not encompass the entirety of an athlete’s life, and general stress may also stem from academics, interpersonal relationships, and future career development. Therefore, the findings of this study cannot be narrowed down to the conclusion that coach support reduces competition stress, nor can they distinguish which source—family, friends, or significant others—is more critical. A more prudent interpretation is that there is a consistent negative correlation between overall perceived support and overall stress experience. Since cross-sectional data cannot determine causality, athletes experiencing higher stress may also find it more difficult to perceive or utilize the support available to them.

Perceived general stress was significantly and positively associated with athlete burnout, consistent with the general trend in domestic and international research on stress and athlete burnout (Lin et al., 2022; Gustafsson and Skoog, 2012; Olsson et al., 2022; Chen et al., 2011; Wu et al., 2016). When athletes consistently feel that life’s demands are unmanageable or overwhelming, emotional and physical exhaustion may become more pronounced, and their sense of accomplishment and evaluation of the value of sports may also be affected. At the same time, the PSS-14 measures general stress perception over the past month, rather than sport-specific stressors such as training volume, competition demands, injury risks, or selection outcomes. Therefore, this association cannot be specifically attributed to any one type of competitive stress, nor can it be used to determine which stress source should be prioritized for intervention. Future studies may simultaneously incorporate general stress, sport-specific stress, and objective training load to compare the relationships between different stress indicators and the various dimensions of burnout.

Perceived social support was found to have a significant negative overall association with athlete burnout, which is generally consistent with theoretical expectations regarding social support as a psychosocial resource and with existing research (Pacewicz et al., 2019; Shang and Yang, 2021; Gray et al., 2024; Murray et al., 2023; Zhao et al., 2022; Wu et al., 2016). Athletes who feel they can obtain understanding and assistance tend to report lower levels of exhaustion, less devaluation of sport, and a more positive sense of accomplishment. This study did not independently measure support from coaches, teammates, family, or the organization, nor did it assess potential processes such as psychological resilience, the need for belonging, or coping strategies. Therefore, the overall association cannot be further interpreted as the direct effect of a specific source of support or a particular psychological mechanism. This finding primarily indicates a consistent link between perceived social support and athlete burnout; specific sources and conditions of this effect still require more refined measurement designs.

### 5.2 Statistical indirect effects of general perceived stress

The Bootstrap results showed that the statistical indirect effect between perceived social support and athlete burnout was significant, whereas the direct association was not significant after including general stress perception, forming an “indirect-only” pattern. According to traditional statistical classification, this pattern can be termed “full mediation,” but the term “full” merely describes the statistical status of the direct path in the model and does not imply that general stress perception is the sole underlying mechanism. Previous studies have observed similar indirect structures among stress resources, perceived stress, and burnout (Gustafsson and Skoog, 2012; Olsson et al., 2022; Chen et al., 2011; Raedeke and Smith, 2004); however, when all three variables are measured simultaneously, the temporal sequence remains unconfirmed, and the possibility that athlete burnout may, in turn, influence perceived support or stress experience cannot be ruled out. Cross-sectional indirect effects may also be influenced by common methods, unmeasured confounders, and alternative models; therefore, they are best understood as statistical relationships consistent with theoretical expectations.

This study did not measure potential mechanisms such as intrinsic motivation, body image, technical feedback from coaches, or support from team physicians; therefore, these variables cannot be used to explain the statistical indirect effects or infer other direct pathways. Existing research suggests that factors such as different sources of support, organizational stress, individual coping resources, and perfectionism may all be associated with burnout (Hobfoll, 1989; Hobfoll et al., 2018; Lazarus and Folkman, 1984; Cohen and Wills, 1985; Raedeke and Smith, 2004). A reasonable direction for future research would be to measure these variables separately in a longitudinal design and compare various temporal sequences and alternative models. Only when variables are measured at multiple time points and the theoretical sequence is clearly defined can mediating processes be discussed with greater certainty.

The relationship between social support, general stress perception, and athlete burnout may also be influenced by sport type, training environment, competitive level, and individual developmental stage. Individual and team sports have different interactive structures, and athletes’ perceptions of significant others may also differ (Raedeke and Smith, 2001; Chen and Zhou, 2007). This study could only obtain the overall distribution of sports from team recruitment records; it was unable to accurately match specific sports to each participant. Consequently, it could not test for sport-specific differences nor determine whether the quality, timing, or specific sources of support are more critical than the overall level of support. These issues require further examination in studies that retain individual sport information and team identifiers.

Resource Conservation Theory and the Cognitive Transaction Stress Model can provide complementary explanations for the statistical relationships among the three variables (Hobfoll, 1989; Hobfoll et al., 2018; Lazarus and Folkman, 1984; Cohen and Wills, 1985; Raedeke and Smith, 2004). The former emphasizes the relationship between the perception of interpersonal resources represented by social support and burnout, while the latter highlights the link between general stress appraisal and burnout; the stress buffering perspective, in turn, provides a theoretical basis for the negative association between social support and perceived stress. The findings of this study support the existence of directionally consistent cross-sectional relationships among these variables but do not validate a closed causal chain. Its theoretical value lies in distinguishing athlete burnout from short-term mental fatigue, clarifying that the PSS-14 measures general stress, and testing the stability of statistical patterns in sensitivity analyses that control for multiple sample characteristics.

### 5.3 Theoretical implications and research boundaries

Conceptual boundaries are a prerequisite for interpreting the results of this study. The Athlete Burnout Questionnaire measures athlete burnout, not short-term cognitive fatigue (Raedeke and Smith, 2001; Chen and Zhou, 2007); the PSS-14 measures general perceived stress over the past month, not stress specific to training and competition (Cohen et al., 1983; Yang and Huang, 2003); and the Perceived Social Support Scale reflects overall perceived support, not specific sources of support (Zimet et al., 1988). Limiting theoretical interpretations to the actual scope covered by the scales helps avoid directly equating general social support, general stress, and athlete burnout with overly specific mechanisms in competitive contexts.

The results suggest that resource and stress appraisals may be associated with athlete burnout from different perspectives. Perceived social support reflects athletes’ perception of external interpersonal resources, while general stress perception reflects their overall evaluation of recent life demands. Statistical indirect effects indicate that the two variables can form a directionally consistent association structure within the model; however, they do not prove that one variable necessarily exerts its influence through the other (Gustafsson and Skoog, 2012; Olsson et al., 2022; Chen et al., 2011; Raedeke and Smith, 2004). This interpretation preserves the insights offered by Resource Conservation Theory and the Cognitive Transaction Stress Model while avoiding the exaggeration of cross-sectional statistical findings into established psychological mechanisms.

Macro-level policies and professional service systems provide the institutional framework for safeguarding athletes’ mental health (Pacewicz et al., 2019; Shang and Yang, 2021; Gray et al., 2024; Murray et al., 2023; Zhao et al., 2022; Wu et al., 2016), but institutional provision does not equate to individuals actually receiving and perceiving effective support. Whether a sports team has established a support mechanism, whether athletes genuinely feel supported, how general stress levels fluctuate, and whether burnout subsequently develops all fall under different analytical levels—organizational and individual, respectively. Distinguishing between institutional provision, team environment, and individual psychological processes helps avoid conflating policy objectives, organizational measures, and individual psychological outcomes into an untested causal chain.

### 5.4 Lessons from practice

The findings of this study can serve as a reference for sports teams in addressing athletes’ perceptions of social support, experiences of general stress, and levels of burnout. Research on mental health services for athletes, both domestically and internationally, emphasizes ongoing assessment, professional collaboration, and referral when necessary (Rees and Hardy, 2000; Shang and Yang, 2021; Gray et al., 2024). In practical application, low perceived support, high general stress, and high burnout levels may indicate a need for further understanding of the athlete’s situation; however, subsequent assessments should be conducted by professionally qualified personnel based on interviews, training context, and ongoing observation. Since this study did not examine diagnostic validity, optimal screening timing, or the effectiveness of interventions, it is not appropriate to establish specific score thresholds, fixed assessment timepoints, or intervention dosages based on these findings.

### 5.5 Limitations of the study

This study has several limitations. First, it employed a cross-sectional self-report questionnaire, with the three core variables measured at a single point in time; thus, it was not possible to determine the temporal sequence or direction of causality, nor could reverse relationships be ruled out. Second, the PSS-14 measures general stress rather than sport-specific stress, and the Social Support Scale does not distinguish separately between support from coaches, teammates, family, and organizations; Although some items regarding significant others included examples from sports contexts, the scale’s structure still requires further validation in a sample of competitive athletes. Third, all variables were self-reported by the athletes; common method effects and social desirability may have influenced the strength of correlations. The results of Harman’s one-factor analysis only indicate that no single factor dominated the data and cannot completely rule out common method bias. Fourth, there was an overlap in the age grouping at 23 years, and exact ages were not recorded. Fifth, participants were recruited from 32 training teams; athletes within the same team may share coaches, training schedules, and team atmosphere, but the final analysis file did not include individual `team_id’s, making it impossible to calculate the ICC or employ team clustering for robust inference. If intra-team correlations are strong, the conventional standard error may be underestimated. Sixth, although the sample spans multiple regions and sports, it does not constitute a probability sample of the national athlete population. Recent reviews have similarly pointed out that research on athlete burnout still requires more longitudinal, repeated-measure, and multilevel designs (Gustafsson et al., 2017; Madigan et al., 2022; Glandorf et al., 2025; Dišlere et al., 2025).

## 6 Conclusion

This study examined the relationship among perceived social support, general stress perception, and athlete burnout among 252 competitive athletes. The results showed that perceived social support was significantly negatively correlated with athlete burnout and with general stress perception; general stress perception was significantly positively correlated with athlete burnout. Bootstrap analysis indicated that the statistical indirect effect was significant, while the direct association was not significant after including general stress perception, presenting an “indirect-only” pattern, which can be classified as “full mediation” under traditional classification. Sensitivity analyses adjusting for covariates yielded the same statistical pattern, suggesting that the original relationship remained relatively stable after controlling for gender, team level, sport level, age group, and training characteristics. However, it should be noted that full mediation is merely a statistical classification; cross-sectional data cannot prove that general stress perception mediates the relationship between social support and burnout over time or causally. The conclusions of this study should be limited to the concurrent associations among the three constructs. Future research should employ longitudinal or experimental designs, distinguish between general stress and sport-specific stress, retain information at the team and program levels, and use statistical methods better suited for nested data.

## Data Availability

The raw data supporting the conclusions of this article will be made available by the authors, without undue reservation.
